# Real-World Impact of Blood Pressure Control in Patients With Apparent Treatment-Resistant or Difficult-to-Control Hypertension and Stages 3 and 4 Chronic Kidney Disease

**DOI:** 10.1093/ajh/hpae020

**Published:** 2024-03-04

**Authors:** George Bakris, Cindy Chen, Alicia K Campbell, Veronica Ashton, Lloyd Haskell, Mukul Singhal

**Affiliations:** University of Chicago Medicine, Chicago, Illinois, USA; Janssen Scientific Affairs, LLC, Titusville, New Jersey, USA; Janssen Scientific Affairs, LLC, Titusville, New Jersey, USA; Janssen Scientific Affairs, LLC, Titusville, New Jersey, USA; Janssen Research & Development, LLC, Raritan, New Jersey, USA; Janssen Scientific Affairs, LLC, Titusville, New Jersey, USA

**Keywords:** blood pressure, blood pressure control, chronic kidney disease, health economics, hypertension, real-world analysis, treatment-resistant hypertension

## Abstract

**BACKGROUND:**

Chronic kidney disease (CKD) is a common comorbidity in patients with apparent treatment-resistant hypertension (aTRH). We assessed clinical outcomes, healthcare resource utilization events, and costs in patients with aTRH or difficult-to-control hypertension and stage 3–4 CKD with uncontrolled vs. controlled BP.

**METHODS:**

This retrospective cohort study used linked IQVIA Ambulatory EMR–US and IQVIA PharMetrics Plus claims databases. Adult patients had claims for ≥3 antihypertensive medication classes within 30 days between 01/01/2015 and 06/30/2021, 2 office BP measures recorded 1–90 days apart, ≥1 claim with ICD-9/10-CM diagnosis codes for CKD 3/4, and ≥1 year of continuous enrollment. Baseline BP was defined as uncontrolled (≥130/80 mm Hg) or controlled (<130/80 mm Hg) BP. Outcomes included risk of major adverse cardiovascular events plus (MACE+; stroke, myocardial infarction, heart failure hospitalization), end-stage renal disease (ESRD), healthcare resource utilization events, and costs during follow-up.

**RESULTS:**

Of 3,966 patients with stage 3–4 CKD using ≥3 antihypertensive medications, 2,479 had uncontrolled BP and 1,487 had controlled BP. After adjusting for baseline differences, patients with uncontrolled vs. controlled BP had a higher risk of MACE+ (HR [95% CI]: 1.18 [1.03–1.36]), ESRD (1.85 [1.44–2.39]), inpatient hospitalization (rate ratio [95% CI]: 1.35 [1.28–1.43]), and outpatient visits (1.12 [1.11–1.12]) and incurred higher total medical and pharmacy costs (mean difference [95% CI]: $10,055 [$6,741–$13,646] per patient per year).

**CONCLUSIONS:**

Patients with aTRH and stage 3–4 CKD and uncontrolled BP despite treatment with ≥3 antihypertensive classes had an increased risk of MACE+ and ESRD and incurred greater healthcare resource utilization and medical expenditures compared with patients taking ≥3 antihypertensive classes with controlled BP.

Hypertension is a leading contributor to preventable mortality worldwide and a major cause of cardiovascular and kidney disease, highlighting the need for improved blood pressure (BP) control strategies to reduce the associated burden on individuals and health services.^1–3^ For many patients, hypertension is adequately controlled with a regimen of up to 3 antihypertensive drugs. However, for some patients, BP remains above goal despite concurrent treatment with 3 antihypertensive medications of different classes, or they require 4 or more antihypertensives to achieve target BP. Such cases are termed apparent treatment-resistant hypertension (aTRH).^1,4^ Pooled data from 2009 to 2014 showed that more than 10.3 million adults in the United States had aTRH according to the American College of Cardiology (ACC) and American Heart Association (AHA) 2017 guidelines definition of uncontrolled BP (systolic BP/diastolic BP ≥130/80 mm Hg).^4,5^ When using the older definition of uncontrolled BP (≥140/90 mm Hg), 9.2 million adults met the definition of aTRH.^5,6^

Compared with individuals with nonresistant hypertension, patients with aTRH are at an increased risk for adverse clinical outcomes, have impairments in health-related quality of life, and have an increased risk of cardiovascular mortality.^4,7–10^ There appears to be a close association between aTRH and the development and progression of chronic kidney disease (CKD).^11^ Almost 20% of patients with aTRH have CKD^12^ while, in patients with CKD, up to half meet the definition of aTRH.^13,14^ In patients with CKD, the presence of aTRH is statistically significantly associated with an increased risk of renal events (adjusted hazard ratio [HR], 1.28; 95% confidence interval [CI]: 1.11, 1.46) and cardiovascular complications (i.e., myocardial infarction, stroke, peripheral artery disease, congestive heart failure [HF]; HR, 1.48; 95% CI: 1.28, 1.46).^13^ While the clinical burden of aTRH on patients with CKD has been documented, data evaluating healthcare and economic burden among patients with aTRH and comorbid CKD are limited. The objective of this analysis was to assess clinical outcomes, healthcare resource utilization events, and costs among patients with hypertension using ≥3 antihypertensive medication classes and stage 3/4 CKD, comparing those with uncontrolled vs. controlled BP.

## METHODS

### Data sources

The details of the data sources used in this study have been published previously.^15^ In brief, the study used linked data from 2 US-based databases: the IQVIA Ambulatory EMR–US database and the IQVIA adjudicated health plan claims (PharMetrics Plus) data. If a variable for the same patient was present in both databases, PharMetrics Plus was used as the primary source. These data were made available by IQVIA and used under license for the current study and are not publicly available. Other researchers should contact https://www.iqvia.com.

### Study design and patients

The design of this retrospective cohort study has been published previously (Supplementary Figure S1 online).^15^ For the current analysis, eligible patients had to be ≥18 years of age, concurrently taking ≥3 antihypertensive medication classes within 30 days between 1 January 2015 and 30 June 2021, and have stage 3/4 CKD at baseline based on *International Classification of Diseases, Ninth/Tenth Revision, Clinical Modification* (ICD-9/10-CM) diagnosis codes. The index regimen date was defined as the date when the criterion for concurrent use of ≥3 antihypertensive medications was met. Patients were required to have 2 office-based BP measurements (on different dates ≤90 days apart) between 30 days and 11 months after the index regimen date, and the date of the second measurement was defined as the index date. Patients had to have ≥365 days of continuous health plan enrollment before the index date, which served as the baseline period.

### Definitions and assessments

Baseline systolic and diastolic BP were defined as the mean of the first 2 measurements recorded during office visits on different dates ≤90 days apart between 30 days and 11 months after the index regimen date, an approach consistent with clinical guidelines.^4^ In the primary analyses, uncontrolled BP was defined as ≥130/80 mm Hg, and controlled BP was defined as <130/80 mm Hg, consistent with the 2017 and 2021 ACC/AHA guideline recommendations.^4,16^ We also conducted a sensitivity analysis using the BP <140/90 mm Hg goal as controlled BP and BP ≥140/90 mm Hg as uncontrolled BP per the 2020 International Society of Hypertension (ISH) guidelines and the Seventh Report of the Joint National Committee (JNC 7).^17,18^

The composite major adverse cardiovascular events plus (MACE+) endpoint included hospitalization with a primary diagnosis of stroke, myocardial infarction, or HF during the follow-up period, while end-stage renal disease (ESRD) was defined as being on dialysis for ≥90 days or undergoing kidney transplantation during the follow-up period. Patients were followed until the earliest of the first MACE+ or ESRD event, end of health plan enrollment, or end of available data.

The following healthcare resource utilization events were assessed: hospitalizations and length of stay, emergency room visits, hospital outpatient visits, physician office visits, and pharmacy fills. Healthcare resource utilization costs were categorized as medical costs (hospitalization, emergency room visits, hospital outpatient visits, and primary care or specialist physician office visits), pharmacy costs (pharmacy fills), and overall healthcare costs (medical plus pharmacy costs).

### Ethics

Use of the IQVIA databases was reviewed by the New England Institutional Review Board (IRB) and was determined to be exempt from broad IRB approval, as this study was a retrospective database review that did not involve identified human participants. Confidentiality of patient records was maintained throughout the study. Study reports did not identify individual patients or physicians. The sponsor did not receive patient-identifying information during the study.

### Statistical analysis

Patient demographics and clinical characteristics were assessed using descriptive statistics, including means and SDs for continuous variables and relative frequencies and percentages for categorical variables. Between-group differences were assessed using standardized differences. A standardized difference <10% was considered balanced. For clinical outcomes, incidence rates were calculated as the total number of cases during the follow-up over total follow-up time (years) and reported per 1,000 patient-years (PYs). Multivariable regression models were used for between-group comparisons. Conditional Cox proportional hazard models were used, adjusting for unbalanced covariates, and calculating the HR and corresponding 95% CI. Rates of healthcare resource utilization were calculated as the number of events divided by PYs of observation. Rate ratios (RRs) were used for between-group comparisons. Direct costs were calculated using mean cost per PY, which was calculated by dividing the costs incurred during the follow-up period by PYs of observation. All costs were inflated to 2021 US dollars based on the medical care component of the Consumer Price Index. A generalized linear model with gamma distribution was used to estimate the mean differences, 95% CIs, and *P* values. *P* values <0.05 were considered statistically significant. All analyses were performed using the Instant Health Data Platform (Panalgo, LLC, Boston, MA) and SAS Enterprise 7.15 (SAS Institute, Cary, NC).

## RESULTS

### Patient disposition

**Figure 1.**
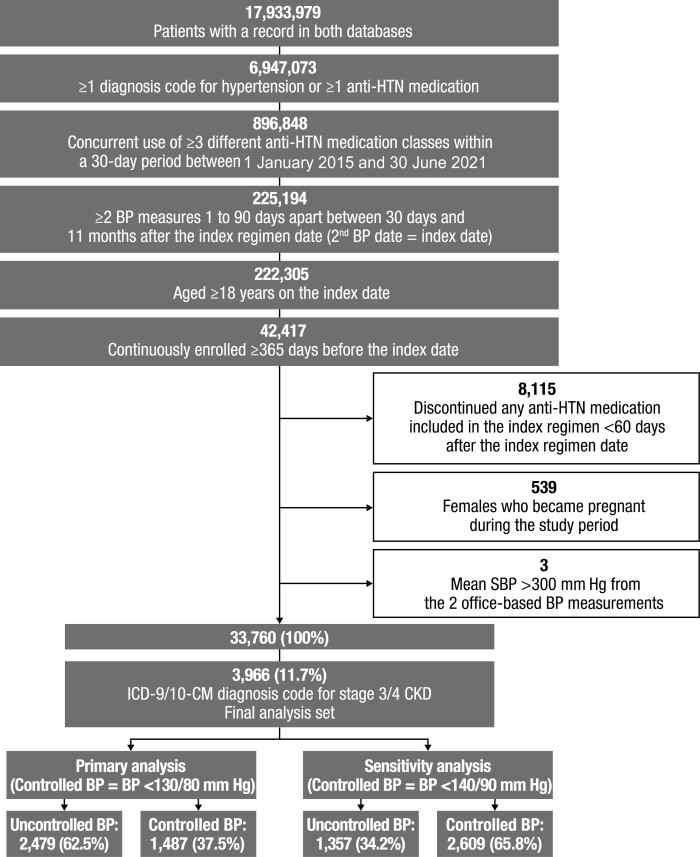
Patient flowchart for IQVIA Ambulatory EMR–US and PharMetrics Plus linked claims. Abbreviations: anti-HTN, antihypertensive; BP, blood pressure; CKD, chronic kidney disease; ICD-9/10-CM, *International Classification of Diseases, Ninth/Tenth Revision, Clinical Modification*; SBP, systolic blood pressure.

Of the approximately 18 million patients who were present in both databases, 3,966 had an ICD-9/10-CM diagnosis code for stage 3/4 CKD and met all other inclusion criteria (Figure 1). Of the 3,966 patients included in the analysis, 2,479 (62.5%) had uncontrolled BP and 1,487 (37.5%) had controlled BP according to the primary analysis definition of uncontrolled BP (≥130/80 mm Hg), while 1,357 (34.2%) had uncontrolled BP and 2,609 (65.8%) had controlled BP according to the sensitivity analysis definition of uncontrolled BP (≥140/90 mm Hg).

### Patient demographic and clinical characteristics during baseline period

There were imbalances (standardized differences ≥10%) in patient characteristics at baseline between those with uncontrolled and controlled BP (Tables 1 and 2 and Supplementary Table S1 online). Patients with uncontrolled BP were younger than those with controlled BP (64.5 vs. 67.5 years), and there was a greater proportion of African American patients in the uncontrolled vs. controlled BP group (15.4% vs. 10.4%). Baseline comorbidities that were less frequently observed in patients with uncontrolled vs. controlled BP included congestive HF (34.1% vs. 42.9%), chronic pulmonary disease (30.5% vs. 38.5%), peripheral vascular disease (25.3% vs. 33.5%), atrial fibrillation (18.5% vs. 29.3%), and myocardial infarction (11.7% vs. 16.3%; Table 2). The mean number of antihypertensive medication classes taken at baseline was higher in the uncontrolled vs. controlled BP group (3.4 vs. 3.3; Table 1). There were no statistically significant differences between the uncontrolled and controlled BP groups in baseline healthcare resource utilization events or costs (Table 2).

**Table 1.** Patient baseline demographic characteristics and antihypertensive medication use (primary analysis population)

	Uncontrolled BP (*n* = 2,479)	Controlled BP (*n* = 1,487)	Standardized difference,^a^ %
Age, y, mean (SD)	64.5 (13.1)	67.5 (13.4)	22.5
Age group, y, *n* (%)			
18–44	170 (6.9)	82 (5.5)	5.6
45–64	1,162 (46.9)	538 (36.2)	21.8
65–74	559 (225)	372 (25.0)	5.8
75–79	246 (9.9)	184 (12.4)	7.8
≥80	342 (13.8)	311 (20.9)	18.9
Men, *n* (%)	1,398 (56.4)	811 (54.5)	3.7
Race, *n* (%)			
White	1,631 (65.8)	1,069 (71.9)	13.2
Black or African American	382 (15.4)	154 (10.4)	15.1
Asian	95 (3.8)	72 (4.8)	5.0
Other	48 (1.9)	27 (1.8)	0.9
Unknown	319 (12.9)	162 (10.9)	6.1
Insurance type, *n* (%)			
Commercial	1,099 (44.3)	569 (38.3)	12.3
Medicare^b^	607 (24.5)	494 (33.2)	19.4
Self-insured	655 (26.4)	362 (24.3)	4.8
Medicaid	117 (4.7)	62 (4.2)	2.7
Unknown/missing	1 (0.04)	0	2.8
US Census region, *n* (%)			
South	1,176 (47.4)	617 (41.5)	12.0
Midwest	503 (20.3)	380 (25.6)	12.5
Northeast	371 (15.0)	218 (14.7)	0.9
West	427 (17.2)	270 (18.2)	2.4
Unknown	2 (0.1)	2 (0.1)	1.6
Number of antihypertensive medication classes^c^			
Mean (SD)	3.4 (0.7)	3.3 (0.6)	20.8
3, *n* (%)	1,670 (67.4)	1,138 (76.5)	20.5
4, *n* (%)	623 (25.1)	283 (19.0)	14.7
≥5, *n* (%)	186 (7.5)	66 (4.4)	13.0

Abbreviation: BP, blood pressure.

^a^Standardized difference with values ≥10% considered statistically significant.

^b^Medicare Part C, Medicare Advantage, and Medicare Supplemental Plans.

^c^Eleven antihypertensive medication classes were included in the study: diuretics (including loop diuretics, thiazide diuretics, potassium-sparing diuretics, and mineralocorticoid receptor antagonists), beta blockers, angiotensin-converting enzyme inhibitors, angiotensin receptor blockers, calcium channel blockers, alpha blockers, alpha-2 receptor agonists, combined alpha and beta blockers, central agonists, peripheral adrenergic inhibitors, and vasodilators.

**Table 2.** Patient baseline clinical characteristics, comorbid conditions, and healthcare resource utilization costs (primary analysis population)

	Uncontrolled BP (*n* = 2,479)	Controlled BP (*n* = 1,487)	Standardized difference,^a^ %
SBP^b^			
Mean (SD)	143.8 (14.4)	118.2 (8.2)	218.8
Median (IQR)	140.5 (17.5)	120.0 (10.0)	
DBP^b^			
Mean (SD)	79.0 (10.1)	68.2 (7.2)	123.3
Median (IQR)	79.6 (13.0)	69.0 (10.5)	
QCI score			
Mean (SD)	3.3 (2.2)	3.7 (2.4)	13.9
Comorbidities ≥10%, *n* (%)			
Hyperlipidemia	2,106 (85.0)	1,293 (87.0)	5.8
Type 2 diabetes mellitus	1,590 (64.1)	887 (59.7)	9.3
Anemia	1,272 (51.3)	727 (48.9)	4.8
Congestive heart failure	846 (34.1)	638 (42.9)	18.1
Chronic pulmonary disease	755 (30.5)	573 (38.5)	17.1
Peripheral vascular disease	628 (25.3)	498 (33.5)	18.0
Atrial fibrillation	458 (18.5)	436 (29.3)	25.6
Cerebrovascular disease	540 (21.8)	364 (24.5)	6.4
Depression	509 (20.5)	355 (23.9)	8.0
Osteoarthritis	457 (18.4)	289 (19.4)	2.6
Anxiety	445 (18.0)	285 (19.2)	3.1
Any malignancy^c^	409 (16.5)	254 (17.1)	1.6
MI	290 (11.7)	242 (16.3)	13.2
Mild liver disease	223 (9.0)	165 (11.1)	7.0
Stroke	240 (9.7)	155 (10.4)	2.5
Possible causes of secondary hypertension, *n* (%)			
Sleep apnea	661 (26.7)	363 (24.4)	5.2
Alcohol use disorder	105 (4.2)	56 (3.8)	2.4
Renal artery stenosis	37 (1.5)	14 (0.9)	5.0
Hyperaldosteronism	13 (0.5)	5 (0.3)	2.9
Cushing syndrome	2 (0.1)	0	4.0
Specific diagnosis code for secondary hypertension	71 (2.9)	22 (1.5)	9.5
BMI, kg/m^2^			
Mean (SD)	33.3 (8.5)	31.7 (8.3)	19.2
Smoking status, *n* (%)			
Smoker	811 (32.7)	535 (36.0)	6.9
Patients with ≥1 healthcare resource utilization event, *n* (%)			
Inpatient hospitalization	1,133 (45.7)	700 (47.1)	2.7
ER visit	727 (29.3)	464 (31.2)	4.1
Hospital outpatient visit	2,385 (96.2)	1,435 (96.5)	1.6
Physician office visit	2,369 (95.6)	1,416 (95.2)	1.6
Number of events PPPY, mean (SD)			
Inpatient hospitalization	1.1 (3.7)	1.1 (1.9)	1.0
ER visit	0.5 (1.4)	0.6 (1.2)	1.6
Hospital outpatient visit	71.8 (82.0)	72.8 (81.0)	1.3
Physician office visit	13.2 (10.2)	13.8 (10.7)	5.5
Pharmacy fill	31.4 (22.4)	33.3 (23.5)	8.3
Length of hospital stay, days, mean (SD)			
All patients	6.6 (16.1)	7.9 (28.2)	6.0
Patients with ≥1 hospitalization	14.4 (21.4)	16.9 (39.2)	7.9
Total medical cost PPPY, USD, mean (SD)	30,959 (56,946)	33,750 (62,107)	4.7
Inpatient hospitalization	17,463 (42,242)	20,444 (52,303)	6.3
ER visit	545 (2,002)	503 (1,687)	2.2
Hospital outpatient visit	11,454 (26,901)	11,216 (22,441)	1.0
Physician office visit	1,359 (1,140)	1,376 (1,246)	1.4
Total pharmacy cost PPPY, USD, mean (SD)	6,276 (17,131)	6,344 (15,326)	0.4
Total cost (medical and pharmacy) PPPY, USD, mean (SD)	37,235 (60,739)	40,094 (64,517)	4.6

Abbreviations: BMI, body mass index; BP, blood pressure; DBP, diastolic blood pressure; ER, emergency room; IQR, interquartile range; MI, myocardial infarction; PPPY, per patient per year; QCI, Quan-Charlson Comorbidity Index; SBP, systolic blood pressure; USD, United States dollars.

^a^Standardized difference with values ≥10% considered statistically significant.

^b^Based on the mean of measurements obtained during 2 office visits on separate days 1–90 days apart.

^c^Includes lymphoma and leukemia; excludes malignant neoplasm of the skin.

### Clinical outcomes

**Figure 2.**
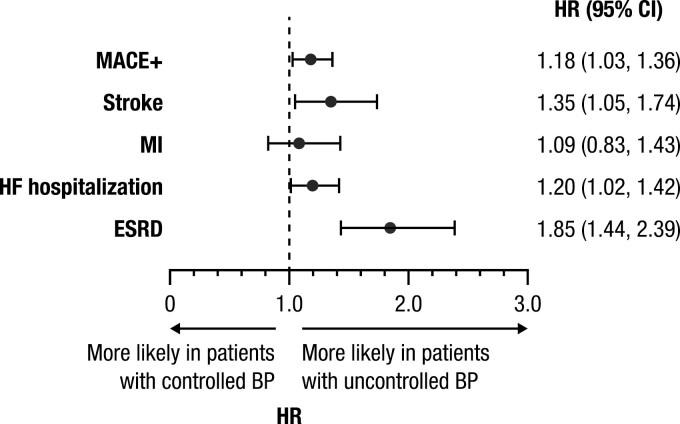
Adjusted clinical outcomes (primary analysis population). (Controlled BP is the reference group. Model was adjusted for age group, race, insurance type, US Census region, baseline comorbidities [Quan-Charlson Comorbidity Index score, congestive heart failure, myocardial infarction, peripheral vascular disease, chronic pulmonary disease, diabetes with chronic complications, atrial fibrillation], BMI category, use of antiplatelets, oral anticoagulants, antiarrhythmic drugs, and antidepressants, and number of index regimen drugs.) Abbreviations: BMI, body mass index; BP, blood pressure; CI, confidence interval; ESRD, end-stage renal disease; HR, hazard ratio; MACE+, major adverse cardiovascular events plus; MI, myocardial infarction.

For MACE+, mean (SD) follow-up time was 2.2 (1.9) years in the uncontrolled BP group and 2.3 (2.0) years in the controlled BP group, while follow-up times for ESRD were 2.3 (2.0) and 2.6 (2.1) years, respectively. The unadjusted rate of the MACE+ composite outcome was 97.3 per 1,000 PY in the uncontrolled BP group and 101.8 per 1,000 PY in the controlled BP group; rates of the individual MACE+ components are shown in Table 3. The unadjusted rate of ESRD was notably higher in the uncontrolled BP group (48.2 per 1,000 PY) compared with the controlled BP group (23.1 per 1,000 PY). After adjusting for baseline differences in demographic and clinical characteristics, compared with patients with controlled BP, patients with uncontrolled BP were at an increased risk of developing MACE+ (HR, 1.18; 95% CI: 1.03, 1.36) and ESRD (HR, 1.85; 95% CI: 1.44, 2.39; Figure 2). MACE+ risk was mainly driven by an increased risk of stroke (HR, 1.35; 95% CI: 1.05, 1.74) and HF hospitalization (HR, 1.20; 95% CI: 1.02, 1.42).

**Table 3.** Unadjusted incidence of clinical outcomes (primary analysis population)

	Uncontrolled BP (*n* = 2,479)		Controlled BP (*n* = 1,487)	
	*n* (%)	Incidence (95% CI)^a^	*n* (%)	Incidence (95% CI)^a^
MACE+	539 (21.7)	97.3 (89.8, 105.5)	348 (23.4)	101.8 (92.1, 112.4)
Stroke	174 (7.0)	28.3 (24.5, 32.8)	101 (6.8)	26.2 (21.6, 31.8)
MI	137 (5.5)	22.0 (18.7, 26.0)	100 (6.7)	26.2 (21.6, 31.8)
HF hospitalization	388 (15.7)	66.9 (60.7, 73.6)	252 (16.9)	70.2 (62.3, 79.1)
ESRD	278 (11.2)	48.2 (43.0, 54.1)	88 (5.9)	23.1 (18.8, 28.4)

Abbreviations: BP, blood pressure; CI, confidence interval; ESRD, end-stage renal disease; HF, heart failure; MACE+, major adverse cardiovascular events plus; MI, myocardial infarction.

^a^Number is per 1,000 person-years at risk.

### Healthcare resource use and costs

**Figure 3.**
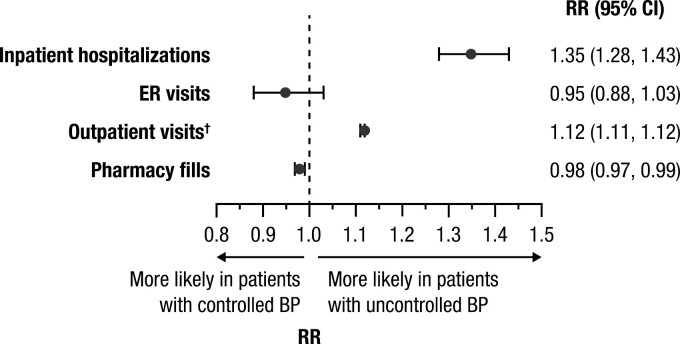
Adjusted healthcare resource utilization events (primary analysis population). (Controlled BP is the reference group. Model was adjusted for age group, race, insurance type, US Census region, baseline comorbidities [Quan-Charlson Comorbidity Index score, congestive heart failure, myocardial infarction, peripheral vascular disease, chronic pulmonary disease, diabetes with chronic complications, atrial fibrillation], BMI category, use of antiplatelets, oral anticoagulants, antiarrhythmic drugs, and antidepressants, and number of index regimen drugs.) ^†^Outpatient visits include hospital outpatient, physician office, and skilled nursing facility visits. Abbreviations: BMI, body mass index; BP, blood pressure; CI, confidence interval; ER, emergency room; RR, rate ratio.

**Figure 4.**
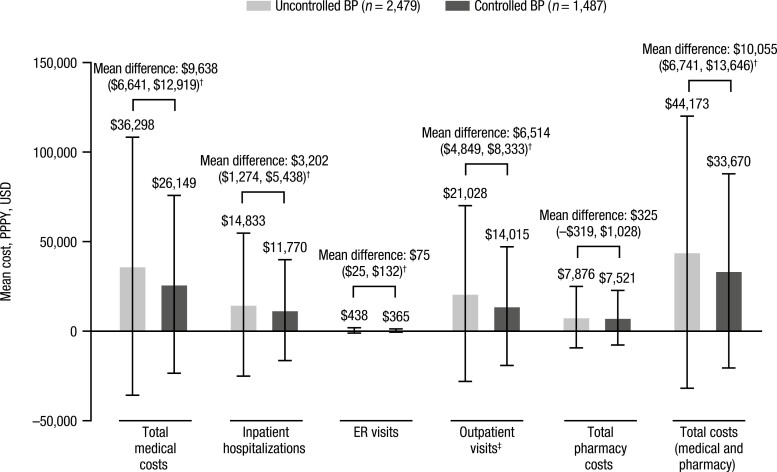
Healthcare resource utilization costs in patients with uncontrolled vs. controlled BP (primary analysis population). (Adjusted healthcare resource utilization cost differences are shown as adjusted estimate [95% CI] using controlled BP as the reference group. Model was adjusted for age group, race, insurance type, US Census region, baseline comorbidities [Quan-Charlson Comorbidity Index score, congestive HF, myocardial infarction, peripheral vascular disease, chronic pulmonary disease, diabetes with chronic complications, atrial fibrillation], BMI category, use of antiplatelets, oral anticoagulants, antiarrhythmic drugs, and antidepressants, and number of index regimen drugs.) ^†^*P* < 0.05. ^‡^Outpatient visits include hospital outpatient, physician office, and skilled nursing facility visits. Abbreviations: BMI, body mass index; BP, blood pressure; CI, confidence interval; ER, emergency room; HF, heart failure; PPPY, per patient per year; USD, United States dollars.

In the primary analysis, patients in the uncontrolled BP group were more likely to have inpatient hospitalizations (adjusted RR, 1.35; 95% CI: 1.28, 1.43) and outpatient visits (adjusted RR, 1.12; 95% CI: 1.11, 1.12) compared with the controlled BP group, while the adjusted RR for emergency room visits was 0.95 (95% CI: 0.88, 1.03; Figure 3). Mean (SD) length of hospital stay among patients with ≥1 hospitalization was 31.84 (59.12) days in patients with uncontrolled BP and 29.09 (43.08) days in those with controlled BP. Adjusted RR for pharmacy fills was 0.98 (95% CI: 0.97, 0.99) for the uncontrolled vs. controlled BP group. Patients in the uncontrolled vs. controlled BP group incurred higher mean total medical costs (adjusted cost difference per patient per year, +$9,638) and mean total pharmacy costs (+$325), resulting in a total additional cost of $10,055 for patients with uncontrolled vs. controlled BP (Figure 4). Total medical costs were driven primarily by outpatient visits (+$6,514) and inpatient hospital admissions (+$3,202). Number of healthcare resource utilization events and mean costs, along with unadjusted RRs for healthcare resource utilization events and costs, are shown in Supplementary Table S2 online.

### Sensitivity analysis

In the sensitivity analysis using BP ≥140/90 mm Hg, imbalances in demographics and comorbidities between the uncontrolled and controlled BP groups at baseline were consistent with those in the primary analyses (Supplementary Tables S3 and S4 online).

Overall, the results for clinical outcomes, healthcare resource utilization events, and costs in the sensitivity analysis were consistent with the primary analysis. In the sensitivity analysis population, mean (SD) follow-up times for MACE+ and ESRD were 2.2 (1.9) and 2.3 (2.0) years, respectively, in the uncontrolled BP group and 2.3 (2.0) and 2.5 (2.1) years, respectively, in the controlled BP group. Unadjusted proportion (rate) of the MACE+ composite outcome was 23.4% (107.8 per 1,000 PY) in the uncontrolled BP group and 21.8% (94.7 per 1,000 PY) in the controlled BP group (Supplementary Table S5 online). The unadjusted proportion and rate of ESRD were higher in the uncontrolled BP group (13.3%, 59.3 per 1,000 PY) compared with the controlled BP group (7.1%, 28.4 per 1,000 PY). After adjusting for baseline differences in demographic and clinical characteristics, patients in the uncontrolled BP group were at a 31% and 82% increased risk of developing MACE+ and ESRD compared with the controlled BP group; increased risk of all MACE+ components was seen in the uncontrolled vs. controlled BP group (Supplementary Figure S2 online). In the sensitivity analysis, patients in the uncontrolled BP group were 26% more likely to have inpatient hospitalizations and 18% more likely to have outpatient visits compared with the controlled BP group, while the adjusted RR for emergency room visits was 1.04 (95% CI: 0.95, 1.13; Supplementary Figure S3 online). Compared with the controlled BP group, the uncontrolled BP group had higher costs for inpatient hospitalizations and outpatient visits, resulting in a $9,073 higher total cost (Supplementary Figure S4 online).

## DISCUSSION

In this retrospective cohort study, we used the IQVIA Ambulatory EMR–US and PharMetrics Plus claims databases to evaluate the healthcare resource utilization burden along with the risk of MACE+ and ESRD in patients with aTRH or difficult-to-control hypertension and stage 3/4 CKD. We found that among CKD stage 3/4 patients, those with hypertension who were using 3 or more antihypertensive treatments but still had uncontrolled BP incurred greater healthcare resource utilization and medical expenditures and had an increased risk of developing MACE+ and ESRD than those with controlled hypertension.

Using the definition of uncontrolled BP in our primary analysis (≥130/80 mm Hg), we found an increase in inpatient hospitalizations (HR, 1.35), an increase in outpatient visits (HR, 1.12), and an increase of approximately $10,000 in total medical and pharmacy costs in patients with uncontrolled BP compared with controlled BP, adjusted for differences in baseline demographic and clinical characteristics. Similarly, in our sensitivity analysis using a BP threshold of ≥140/90 mm Hg, there were increases in inpatient hospitalizations (HR, 1.26) and outpatient visits (HR, 1.18), and total costs increased by approximately $9,000. Thus, our results confirm the healthcare and economic burden of uncontrolled aTRH in patients with stage 3/4 CKD.

aTRH is known to place a burden on healthcare services in terms of resource use and cost. Consistent with our results, in a US population cohort study involving 4,650 participants with hypertension, those with aTRH and uncontrolled BP had a greater mean number of primary care visits (2.77 vs. 2.27 per year; *P* < 0.001), cardiologist visits (0.50 vs. 0.35 per year; *P* = 0.014), and nephrology visits (0.24 vs. 0.07 per year; *P* < 0.001) than those without aTRH. Participants with controlled aTRH had fewer visits compared with those with uncontrolled BP, although the differences were not statistically significant.^19^

Overall, hypertension is one of the top 10 highest health expenditures in the United States, costing an estimated $79 billion in 2016.^20^ The presence of comorbidities adds to the economic burden of hypertension. Data from the 2011–2014 Medical Expenditure Panel Survey estimated mean annual costs of hypertension ranging from $3,914 per patient for those with no comorbidities to $13,920 for those with ≥3 comorbidities; in particular, the presence of comorbid kidney disease increased annual medical expenditure to $14,248 per patient compared with $9,274 for hypertensive patients without kidney disease.^21^ Our results add further evidence of the economic burden of aTRH for patients with kidney disease.

In this analysis, patients in the uncontrolled BP cohort were younger and had a lower incidence of comorbidities (e.g., congestive HF, atrial fibrillation, peripheral vascular disease, chronic pulmonary disease) at baseline compared with patients in the controlled BP cohort, who represent a sicker population. The more controlled BP in this sicker population may be related to more frequent physician visits and better adherence related to management of comorbidities. After adjusting for differences in baseline demographic and clinical characteristics between the uncontrolled and controlled BP groups in the primary analysis, patients with uncontrolled BP (≥130/80 mm Hg) had an 18% increased risk of MACE+ and an 85% increase in ESRD while, in the sensitivity analysis, patients with uncontrolled BP (≥140/90 mm Hg) had a 31% increased risk of MACE+ and an 82% increase in ESRD. Thus, while the absolute number of patients meeting the uncontrolled BP criterion of the sensitivity analysis was reduced relative to the primary analysis, the risk for MACE+ was higher. The greater risk of MACE+ in the sensitivity analysis is consistent with previous findings that elevated BP is associated with higher risk of MACE+^22–24^ and confirms these findings in a population of patients with stage 3/4 CKD.

The increase in ESRD risk in patients with uncontrolled BP compared with controlled BP was similarly high in the primary and sensitivity analyses after adjusting for baseline differences in demographic and clinical characteristics. Our findings are consistent with previous studies showing increased risk of ESRD with elevated BP, even at more stringent BP thresholds.^10,25–27^ For example, in a US cohort study of 316,675 participants without kidney disease at baseline, those with BP of 120–129/80–84 mm Hg had a statistically significant increased risk of 62% for developing ESRD compared with those with BP <120/80 mm Hg, with the magnitude of risk increasing for higher BP categories.^26^ Likewise, in our study population with stage 3/4 CKD, an increased risk for progression of kidney disease to ESRD was evident at the more stringent threshold for uncontrolled BP of ≥130/80 mm Hg in the primary analysis. The results of this study reinforce the potential for earlier and more stringent BP control in reducing the risk of ESRD, particularly in patients with existing kidney disease.

Our findings of increased risk of adverse clinical outcomes in patients with CKD and uncontrolled vs. controlled BP are consistent with those of previous studies. In an analysis of 3,367 patients with CKD enrolled in the Chronic Renal Insufficiency Cohort study, those with aTRH had a 38% higher risk of the composite endpoint of myocardial infarction, stroke, peripheral arterial disease, congestive HF, and all-cause mortality, and a 28% increased risk of renal events, compared with patients without aTRH.^13^ Likewise, in a prospective study involving 436 patients with CKD, aTRH was associated with a 24% higher risk of cardiovascular events and 2.7-fold higher risk of renal events.^28^

There are limitations to this study associated with the analysis of administrative claims data. The data used in this study were limited by the information recorded and translated into structured data elements and may not be generalizable to the entire US population. The results, however, were derived from a large population-based sample of patients with treated hypertension and CKD that reflects current real-world practice. Errors in BP measurement may have resulted in the inaccurate classification of uncontrolled or controlled BP. In addition, patients with stage 3/4 CKD were identified using ICD-9/10-CM codes and not laboratory data, so patients from this group may have been underreported and patients with a low estimated glomerular filtration rate and/or proteinuria may have been missed. Claims data are prone to coding errors and inconsistencies; while statistical methods were used to balance patient groups, the potential for residual confounding could not be eliminated. Claims for medications do not indicate that medications were taken as prescribed. Medication adherence and the prescription of clinically appropriate dosages are important factors in aTRH; neither was assessed in this study. Furthermore, there is overlap in drug classes used to treat hypertension and HF, and the study did not assess whether all medications were prescribed for hypertension or another indication.

Among patients with stage 3/4 CKD, those with uncontrolled aTRH had an increased risk of developing MACE+ and ESRD and incurred greater healthcare resource utilization and medical expenditures than those with controlled hypertension who used 3 or more antihypertensive classes. Controlling BP may subsequently reduce healthcare resource utilization and economic burden in patients with aTRH and stage 3/4 CKD.

## Supplementary Material

hpae020_suppl_Supplementary_Figures_S1-S4_Tables_1-S5

## Data Availability

The data sharing policy of Janssen Pharmaceutical Companies of Johnson & Johnson is available at https://www.janssen.com/clinical-trials/transparency. These data were made available by IQVIA and used under license for the current study and are not publicly available. Other researchers should contact https://www.iqvia.com.
