# Supplementary material for: Real-World Impact of Blood Pressure Control in Patients With Apparent Treatment-Resistant or Difficult-to-Control Hypertension and Stages 3 and 4 Chronic Kidney Disease
Source: Am J Hypertens. 2024 Mar 4;37(6):438–46. doi: 10.1093/ajh/hpae020 (PMC11094384; doi:10.1093/ajh/hpae020)
Supplement: hpae020_suppl_Supplementary_Figures_S1-S4_Tables_1-S5 [file hpae020_suppl_supplementary_figures_s1-s4_tables_1-s5.docx]

**Supplementary Materials**

| **Supplementary Table S1.** Baseline concomitant non-antihypertensive medications (primary analysis population) | | | |  |
| --- | --- | --- | --- | --- |
|  | **Uncontrolled BP (n = 2,479)** | **Controlled BP (n = 1,487)** | **Standardized difference,* %** |  |
| Antiarrhythmic drugs,^†^ n (%) | 2,427 (97.9) | 1,444 (97.1) | 5.1 |  |
| Antiarrhythmic drugs excluding BBs/CCBs | 267 (10.8) | 236 (15.9) | 15.1 |  |
| Lipid-lowering drugs, n (%) |  |  |  |  |
| Statins | 1,813 (73.1) | 1,141 (76.7) | 8.3 |  |
| PCSK9 inhibitors | 1 (0.04) | 1 (0.1) | 1.2 |  |
| Other lipid-lowering drugs | 541 (21.8) | 309 (20.8) | 2.5 |  |
| Opioids, n (%) | 1,407 (56.8) | 864 (58.1) | 2.7 |  |
| Antidiabetic drugs, n (%) | 1,427 (57.6) | 794 (53.4) | 8.4 |  |
| Noninsulin antidiabetic drugs | 1,070 (43.2) | 614 (41.3) | 3.8 |  |
| Insulin | 886 (35.7) | 465 (31.3) | 9.5 |  |
| Antidepressants, n (%) | 786 (31.7) | 582 (39.1) | 15.6 |  |
| Systemic corticosteroids, n (%) | 878 (35.4) | 573 (38.5) | 6.5 |  |
| Oral anticoagulants, n (%) | 410 (16.5) | 357 (24.0) | 18.7 |  |
| Direct oral anticoagulants | 192 (7.7) | 160 (10.8) | 10.4 |  |
| Warfarin/vitamin K antagonists | 251 (10.1) | 225 (15.1) | 15.1 |  |
| Anxiolytics, n (%) | 547 (22.1) | 345 (23.2) | 2.7 |  |
| Platelet aggregation inhibitors, n (%) | 442 (17.8) | 330 (22.2) | 10.9 |  |
| NSAIDs, n (%) | 349 (14.1) | 212 (14.3) | 0.5 |  |
| Antineoplastic drugs, n (%) | 196 (7.9) | 98 (6.6) | 5.1 |  |
| Stimulants,^‡^ n (%) | 42 (1.7) | 27 (1.8) | 0.9 |  |
| Antimigraine drugs, n (%) | 43 (1.7) | 22 (1.5) | 2.0 |  |
| Weight-loss drugs,^§^ n (%) | 22 (0.9) | 5 (0.3) | 7.1 |  |
| *Standardized difference with values ≥10% considered statistically significant.  ^†^Includes sodium channel blockers (disopyramide, quinidine, and flecainide), all BBs, potassium channel blockers (amiodarone, dronedarone, and sotalol), and all CCBs.  ^‡^Includes amphetamine, caffeine, armodafinil, atomoxetine, methylphenidate, methamphetamine, dextroamphetamine, dexmethylphenidate, doxapram, and modafinil.  ^§^Includes phentermine, phendimetrazine, orlistat, benzphetamine, bupropion/naltrexone, diethylpropion, and phentermine/topiramate.  BB, beta blocker; BP, blood pressure; CCB, calcium channel blocker; NSAID, nonsteroidal anti-inflammatory drug; PCSK9, proprotein convertase subtilisin/kexin type 9 serine protease; SD, standard deviation. | | | | |

| **Supplementary Table S2.** Unadjusted healthcare resource utilization events and costs (primary analysis population) | | |  |
| --- | --- | --- | --- |
|  | **Uncontrolled BP (n = 2,479)** | **Controlled BP (n = 1,487)** | |
| **Number of events, PPPY, mean (SD)** |  |  | |
| Inpatient hospitalization | 1.28 (6.5) | 0.80 (1.4) | |
| ER visit | 0.5 (1.1) | 0.49 (1.0) | |
| Outpatient visits* | 104.8 (122.1) | 86.0 (95.5) | |
| Pharmacy fill | 33.4 (20.8) | 34.1 (22.1) | |
| **All-cause costs, PPPY, USD mean (SD)** |  |  | |
| Total medical cost | $36,298 ($72,014) | $26,149 ($49,637) | |
| Inpatient hospitalization | $14,833 ($39,888) | $11,770 ($28,166) | |
| ER | $438 ($1,442) | $365 ($891) | |
| Outpatient visits* | $21,028 ($49,044) | $14,015 ($33,164) | |
| Total pharmacy cost | $7,876 ($17,159) | $7,521 ($15,235) | |
| Total cost (medical + pharmacy) | $44,173 ($75,967) | $33,670 ($54,209) | |
| *Outpatient visits include hospital outpatient, physician office, and skilled nursing facility visits.  BP, blood pressure; ER, emergency room; PPPY, per patient per year; SD, standard deviation; USD, United States dollars. | | |  |

| **Supplementary Table S3.** Patient baseline demographic characteristics (sensitivity analysis population) | | | |
| --- | --- | --- | --- |
|  | **Uncontrolled BP (n = 1,357)** | **Controlled BP (n = 2,609)** | **Standardized difference,* %** |
| Age, y, mean (SD) | 64.3 (13.3) | 66.3 (13.3) | 15.2 |
| Age group, n (%) |  |  |  |
| 18-44 y | 94 (6.9) | 158 (6.1) | 3.5 |
| 45-64 y | 639 (47.1) | 1,061 (40.7) | 13.0 |
| 65-74 y | 287 (21.1) | 644 (24.7) | 8.4 |
| 75-79 y | 156 (11.5) | 274 (10.5) | 3.2 |
| ≥80 y | 181 (13.3) | 472 (18.1) | 13.1 |
| Men, n (%) | 778 (57.3) | 1,431 (54.8) | 5.0 |
| Race, n (%) |  |  |  |
| White | 849 (62.6) | 1,851 (70.9) | 17.9 |
| Black or African American | 242 (17.8) | 294 (11.3) | 18.7 |
| Asian | 55 (4.1) | 112 (4.3) | 1.2 |
| Other | 23 (1.7) | 52 (2.0) | 2.2 |
| Unknown | 186 (13.7) | 295 (11.3) | 7.3 |
| Insurance type, n (%) |  |  |  |
| Commercial | 617 (45.5) | 1,051 (40.3) | 10.5 |
| Medicare^†^ | 317 (23.4) | 784 (30.0) | 15.2 |
| Self-insured | 357 (26.3) | 660 (25.3) | 2.3 |
| Medicaid | 66 (4.9) | 113 (4.3) | 2.5 |
| Unknown/missing | 0 | 1 (0.04) | 2.8 |
| US Census region, n (%) |  |  |  |
| South | 667 (49.2) | 1,126 (43.2) | 12.1 |
| Midwest | 252 (18.6) | 631 (24.2) | 13.7 |
| Northeast | 207 (15.3) | 382 (14.6) | 1.7 |
| West | 230 (16.9) | 467 (17.9) | 2.5 |
| Unknown | 1 (0.1) | 3 (0.1) | 1.4 |
| *Standardized difference with values ≥10% considered statistically significant.  ^†^Medicare Part C, Medicare Advantage, and Medicare Supplemental Plans.  BP, blood pressure; SD, standard deviation. | | | |

| **Supplementary Table S4.** Patient baseline clinical characteristics, comorbid conditions, concomitant medications, and healthcare resource utilization events and costs (sensitivity analysis population) | | | |
| --- | --- | --- | --- |
|  | **Uncontrolled BP (n = 1,357)** | **Controlled BP (n =** **2,609)** | **Standardized difference,* %** |
| SBP^†^ |  |  |  |
| Mean (SD) | 153.2 (12.5) | 124.3 (10.0) | 255.4 |
| Median (IQR) | 150.0 (15.5) | 125.0 (14.0) |  |
| DBP^†^ |  |  |  |
| Mean (SD) | 81.3 (11.2) | 71.6 (8.5) | 97.3 |
| Median (IQR) | 81.0 (14.5) | 72.0 (12.0) |  |
| QCI score |  |  |  |
| Mean (SD) | 3.4 (2.3) | 3.5 (2.3) | 4.4 |
| Comorbidities ≥10%, n (%) |  |  |  |
| Hyperlipidemia | 1,145 (84.4) | 2,254 (86.4) | 5.7 |
| Type 2 diabetes mellitus | 873 (64.3) | 1,604 (61.5) | 5.9 |
| Anemia | 731 (53.9) | 1,268 (48.6) | 10.6 |
| Congestive heart failure | 499 (36.8) | 985 (37.8) | 2.0 |
| Chronic pulmonary disease | 408 (30.1) | 920 (35.3) | 11.1 |
| Peripheral vascular disease | 334 (24.6) | 792 (30.4) | 12.9 |
| Depression | 250 (18.4) | 614 (23.5) | 12.6 |
| Cerebrovascular disease | 291 (21.4) | 613 (23.5) | 4.9 |
| Atrial fibrillation | 235 (17.3) | 659 (25.3) | 19.5 |
| Anxiety | 211 (15.5) | 519 (19.9) | 11.4 |
| Osteoarthritis | 231 (17.0) | 515 (19.7) | 7.0 |
| Any malignancy^‡^ | 226 (16.7) | 437 (16.7) | 0.3 |
| Myocardial infarction | 153 (11.3) | 379 (14.5) | 9.7 |
| Mild liver disease | 116 (8.5) | 272 (10.4) | 6.4 |
| Stroke | 132 (9.7) | 263 (10.1) | 1.2 |
| Possible causes of secondary hypertension, n (%) |  |  |  |
| Sleep apnea | 346 (25.5) | 678 (26.0) | 1.1 |
| Alcohol use disorder | 56 (4.1) | 105 (4.0) | 0.5 |
| Renal artery stenosis | 20 (1.5) | 31 (1.2) | 2.5 |
| Hyperaldosteronism | 9 (0.7) | 9 (0.3) | 4.5 |
| Cushing syndrome | 1 (0.1) | 1 (0.04) | 1.5 |
| Specific diagnosis code for secondary hypertension | 46 (3.4) | 47 (1.8) | 10.0 |
| BMI, kg/m^2^ |  |  |  |
| Mean (SD) | 33.3 (8.8) | 32.35 (8.3) | 11.3 |
| Median (IQR) | 32.0 (10.4) | 30.4 (9.2) |  |
| Smoking status, n (%) |  |  |  |
| Smoker | 420 (31.0) | 926 (35.5) | 9.7 |
| Medication use |  |  |  |
| Antiarrhythmic drugs,^§^ n (%) | 1,333 (98.2) | 2,538 (97.3) | 6.4 |
| Antiarrhythmic drugs excluding BBs/CCBs | 152 (11.2) | 351 (13.5) | 6.9 |
| Lipid-lowering drugs, n (%) |  |  |  |
| Statins | 996 (73.4) | 1,958 (75.0) | 3.8 |
| PCSK9 inhibitors | 1 (0.1) | 1 (0.04) | 1.5 |
| Other lipid-lowering drugs | 274 (20.2) | 576 (22.1) | 4.6 |
| Opioids, n (%) | 755 (55.6) | 1,516 (58.1) | 5.0 |
| Antidiabetic drugs, n (%) | 791 (58.3) | 1,430 (54.8) | 7.0 |
| Noninsulin antidiabetic drugs | 582 (42.9) | 1,102 (42.2) | 1.3 |
| Insulin | 506 (37.3) | 845 (32.4) | 10.3 |
| Antidepressants, n (%) | 390 (28.7) | 978 (37.5) | 18.7 |
| Systemic corticosteroids, n (%) | 477 (35.2) | 974 (37.3) | 4.5 |
| Anxiolytics, n (%) | 286 (21.1) | 606 (23.2) | 5.2 |
| NSAIDs, n (%) | 190 (14.0) | 371 (14.2) | 0.6 |
| Oral anticoagulants, n (%) | 208 (15.3) | 559 (21.4) | 15.8 |
| Direct oral anticoagulants | 92 (6.8) | 260 (10.0) | 11.5 |
| Warfarin/vitamin K antagonists | 134 (9.9) | 342 (13.1) | 10.2 |
| Platelet aggregation inhibitors, n (%) | 254 (18.7) | 518 (19.9) | 2.9 |
| Antineoplastic drugs, n (%) | 106 (7.8) | 188 (7.2) | 2.3 |
| Stimulants,^¶^ n (%) | 15 (1.1) | 54 (2.1) | 7.7 |
| Antimigraine drugs, n (%) | 16 (1.2) | 49 (1.9) | 5.7 |
| Weight-loss drugs, ^\|\|\|\|^ n (%) | 10 (0.7) | 17 (0.7) | 1.0 |
| Patients with ≥1 healthcare resource utilization event, n (%) |  |  |  |
| Inpatient hospitalization | 628 (46.3) | 1,205 (46.2) | 0.2 |
| ER visit | 416 (30.7) | 775 (29.7) | 2.1 |
| Hospital outpatient visit | 1,305 (96.2) | 2,515 (96.4) | 1.2 |
| Physician office visit | 1,296 (95.5) | 2,489 (95.4) | 0.5 |
| Number of events PPPY, mean (SD) |  |  |  |
| Inpatient hospitalization | 1.2 (4.6) | 1.1 (2.0) | 2.8 |
| ER visit | 0.6 (1.3) | 0.5 (1.3) | 2.3 |
| Hospital outpatient visit | 71.2 (76.0) | 72.7 (84.4) | 1.8 |
| Physician office visit | 13.0 (10.0) | 13.6 (10.6) | 5.6 |
| Pharmacy fill | 30.4 (21.4) | 33.0 (23.5) | 11.4 |
| Length of hospital stay, days, mean (SD) |  |  |  |
| All patients | 6.3 (15.2) | 7.5 (24.1) | 5.8 |
| Patients with ≥1 hospitalization | 13.7 (20.0) | 16.2 (33.4) | 9.2 |
| Total medical cost PPPY, $, mean (SD) | 29,762 (51,084) | 33,172 (62,618) | 6.0 |
| Inpatient hospitalization | 16,854 (39,275) | 19,479 (49,527) | 5.9 |
| ER visit | 541 (1,916) | 523 (1,877) | 0.9 |
| Hospital outpatient visit | 10,931 (23,089) | 11,591 (26,405) | 2.7 |
| Physician office visit | 1,320 (1,105) | 1,389 (1,218) | 5.9 |
| Total pharmacy cost PPPY, mean (SD) | 5,846 (10,565) | 6,539 (18,827) | 4.5 |
| Total cost (medical and pharmacy) PPPY, mean (SD) | 35,607 (53,079) | 39,711 (66,402) | 6.8 |
| *Standardized difference with values ≥10% considered statistically significant.  ^†^Based on the mean of measurements obtained during 2 office visits on separate days 1-90 days apart.  ^‡^Includes lymphoma and leukemia; excludes malignant neoplasm of the skin.  ^§^Includes sodium channel blockers (disopyramide, quinidine, and flecainide), all BBs, potassium channel blockers (amiodarone, dronedarone, and sotalol), and all CCBs.  ^¶\|^Includes amphetamine, caffeine, armodafinil, atomoxetine, methylphenidate, methamphetamine, dextroamphetamine, dexmethylphenidate, doxapram, and modafinil.  ^\|\|\|\|^Includes phentermine, phendimetrazine, orlistat, benzphetamine, bupropion/naltrexone, diethylpropion, and phentermine/topiramate.  BB, beta blocker; BMI, body mass index; BP, blood pressure; CCB, calcium channel blocker; DBP, diastolic blood pressure; ER, emergency room; IQR, interquartile range; NSAID, nonsteroidal anti-inflammatory drug; PCSK9, proprotein convertase subtilisin/kexin type 9 serine protease; PPPY, per patient per year; QCI, Quan-Charlson Comorbidity Index; SBP, systolic blood pressure; SD, standard deviation. | | | |

| **Supplementary Table S5.** Unadjusted incidence of clinical outcomes (sensitivity analysis population) | | | | | |
| --- | --- | --- | --- | --- | --- |
|  | **Uncontrolled BP (n = 1,357)** | | **Controlled BP* (n = 2,609)** | |  |
|  | **n (%)** | **Incidence rate**  **per 1000 PY**  **(95% CI)** | **n (%)** | **Incidence rate**  **per 1000 PY**  **(95% CI)** |  |
| MACE+ | 318 (23.4) | 107.8  (97.1, 119.6) | 569 (21.8) | 94.7  (87.6, 102.4) |  |
| Stroke | 103 (7.6) | 30.89  (25.5, 37.4) | 172 (6.6) | 25.8  (22.3, 29.9) |  |
| MI | 88 (6.5) | 26.3  (21.4, 32.3) | 149 (5.7) | 22.3  (19.0, 26.1) |  |
| HF hospitalization | 228 (16.8) | 73.4  (64.8, 83.2) | 412 (15.8) | 65.5  (59.7, 72.0) |  |
| ESRD | 181 (13.3) | 59.3  (51.5, 68.3) | 185 (7.1) | 28.4  (24.6, 32.7) |  |
| *SBP <140 mmHg and DBP <90 mmHg.  BP, blood pressure; CI, confidence interval; ESRD, end-stage renal disease; HF, heart failure; MACE+, major adverse cardiovascular event plus; MI, myocardial infarction; PY, patient year. | | | | | |

**Supplementary Figure S1.** Study design

**
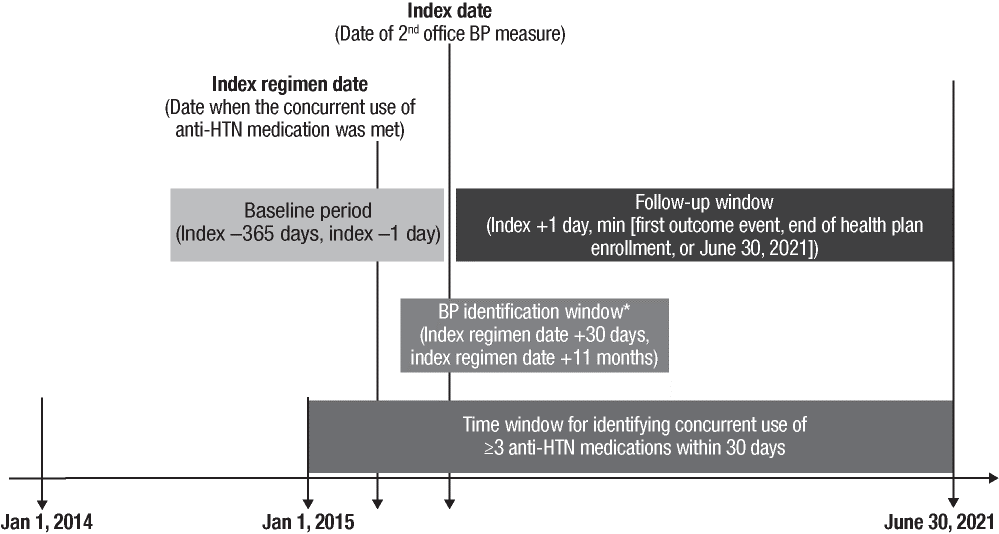
**

*The 2 office-based BP measurements must be on distinct dates and 1 to 90 days apart.

anti-HTN, antihypertensive; BP, blood pressure.

Figure reproduced with permission from Bakris G, Chen C, Campbell AK, Ashton V, Haskell L, Singhal M. Association of uncontrolled blood pressure in apparent treatment-resistant hypertension with increased risk of major adverse cardiovascular events plus. J Clin Hypertens (Greenwich). 2023;25(8):737-747. ©2023 Janssen Scientific Affairs LLC. *The Journal of Clinical Hypertension* published by Wiley Periodicals LLC.

**Supplementary Figure S2.** Adjusted clinical outcomes (sensitivity analysis population)*


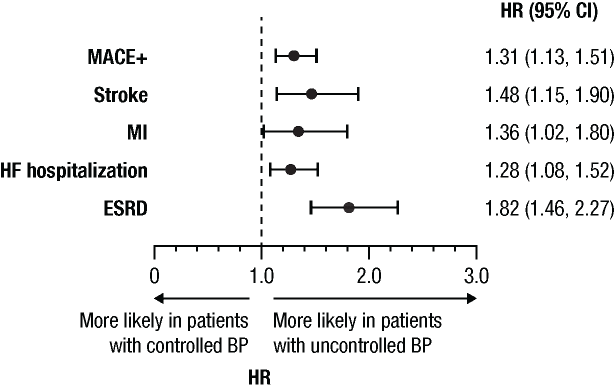


*Controlled BP is the reference group. Model was adjusted for age, race, insurance type, US Census region, baseline comorbidities (peripheral vascular disease, chronic pulmonary disease, diabetes with chronic complications, atrial fibrillation, depression, anxiety), BMI, and use of oral anticoagulants and antidepressants.

BMI, body mass index; BP, blood pressure; CI, confidence interval; ESRD, end-stage renal disease; HF, heart failure; HR, hazard ratio; MACE+, major adverse cardiovascular event plus; MI myocardial infarction.

**Supplementary Figure S3.** Adjusted healthcare resource utilization events (sensitivity analysis population)*


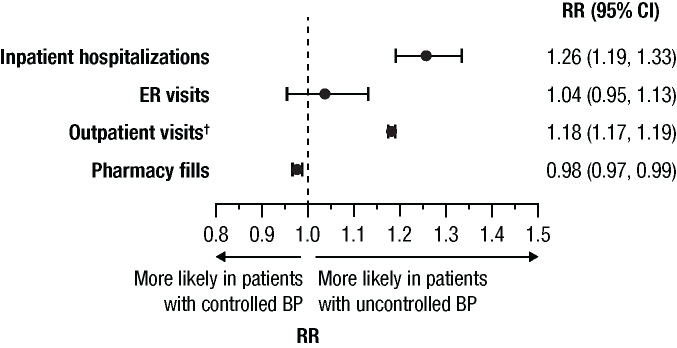


*Controlled BP is the reference group. Model was adjusted for age, race, insurance type, US Census region, baseline comorbidities (peripheral vascular disease, chronic pulmonary disease, diabetes with chronic complications, atrial fibrillation, depression, anxiety), BMI, and use of oral anticoagulants and antidepressants.

^†^Outpatient visits include hospital outpatient, physician office, and skilled nursing facility visits.

BMI, body mass index; BP, blood pressure; CI, confidence interval; ER, emergency room; RR, rate ratio.

**Supplementary Figure S4.** Healthcare resource utilization costs in patients with uncontrolled versus controlled BP* (sensitivity analysis)

**
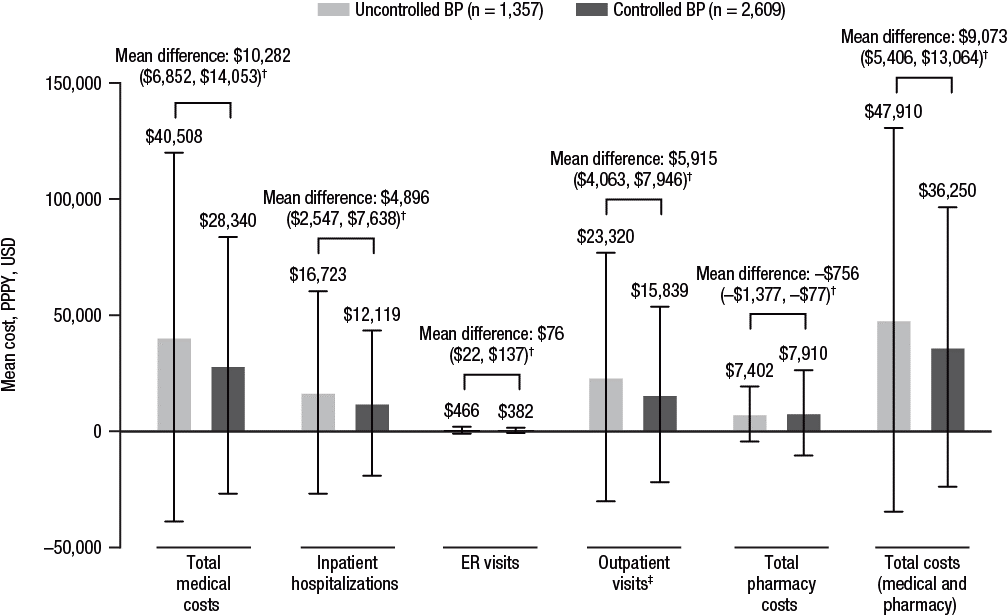
**

*Adjusted healthcare resource utilization cost differences are shown as adjusted estimate (95% CI) using controlled BP as the reference group. Model was adjusted for age, race, insurance type, US Census region, baseline comorbidities (peripheral vascular disease, chronic pulmonary disease, diabetes with chronic complications, atrial fibrillation, depression, anxiety), BMI, and use of oral anticoagulants and antidepressants.

^†^*P* <0.05.

^‡^Outpatient visits include hospital outpatient, physician office, and skilled nursing facility visits.

BMI, body mass index; BP, blood pressure; CI, confidence interval; ER, emergency room; PPPY, per patient per year; SD, standard deviation; USD, United States dollars.
